# An efficient strategy for producing RNA‐free Nucleocapsid protein of SARS‐CoV‐2 for biochemical and structural investigations

**DOI:** 10.1002/2211-5463.70064

**Published:** 2025-06-09

**Authors:** Shweta Singh, Gagan D. Gupta

**Affiliations:** ^1^ Protein Crystallography Section Bhabha Atomic Research Centre Mumbai India; ^2^ Homi Bhabha National Institute Mumbai India

**Keywords:** electrophoretic mobility shift assay, fluorescence polarization, Nucleocapsid protein, SARS‐CoV‐2, Thioredoxin fusion

## Abstract

The SARS‐CoV‐2 Nucleocapsid (N) protein plays a crucial role in genome packaging, replication, transcription, and pathogenesis, making it a promising target for antiviral drug development. However, its large intrinsically disordered regions and propensity to form RNA condensates pose significant challenges for recombinant expression and purification. In this study, we successfully expressed and purified full‐length N protein with a cleavable N‐terminal Thioredoxin (Trx) fusion to enhance solubility and stability. The acidic Trx tag helped in the efficient binding of basic N protein to an anion‐exchange column, enabling complete removal of bound RNA. Through a four‐step process—immobilized metal affinity chromatography (IMAC), anion exchange, TEV protease‐mediated tag cleavage followed by a second IMAC to remove cleaved fragments, and final polishing by size‐exclusion chromatography (SEC)—we obtained highly homogeneous, RNA‐free N protein. A single well‐defined peak on SEC and dynamic light scattering confirmed the homogeneity of the purified protein. Electrophoretic mobility shift assays revealed strong RNA‐binding activity, as a nearly complete RNA shift was observed at N protein concentrations as low as 0.25 μm. Fluorescence polarization assays further quantified RNA‐binding affinity, yielding a dissociation constant of ~28 nm. These results establish an effective strategy for obtaining nucleic acid‐free N protein suitable for biochemical and structural studies. Ultimately, this work provides a foundation for high‐resolution structural investigations and the development of novel antiviral therapeutics targeting the N protein to combat COVID‐19.

## Graphical abstract

Cleavable N‐terminal Thioredoxin fusion enabled soluble expression and purification of otherwise insoluble SARS‐CoV‐2 Nucleocapsid (N) protein. A four‐step purification strategy yielded highly homogeneous, RNA‐free N protein. Binding assays showed high RNA affinity (*K*_d_ ~ 28 nm). The study will facilitate high‐resolution structural studies of N protein, advancing antiviral drug discovery against COVID‐19.

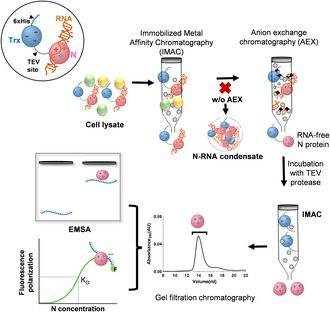

**Abbreviations**
AEX: anion‐exchange chromatography
COVID‐19: coronavirus disease 2019
DLS: dynamic light scattering
EMSA: electrophoretic mobility shift assay
IDR: intrinsically disordered region
IMAC: immobilized metal affinity chromatography
LLPS: liquid–liquid phase separation
N: Nucleocapsid
N‐CTD: C‐terminal domain of N protein
N‐NTD: N‐terminal domain of N protein
SARS‐CoV‐2: severe acute respiratory syndrome coronavirus 2
SEC: size‐exclusion chromatography
Trx: Thioredoxin

Coronavirus disease 2019 (COVID‐19), caused by the severe acute respiratory syndrome coronavirus 2 (SARS‐CoV‐2), continues to pose a global health threat [1]. Despite the widespread rollout of vaccines and therapeutic interventions, the virus remains widely circulating, contributing to both acute infections and chronic postviral complications, such as Long COVID [2, 3]. Long COVID, or Post‐COVID syndrome, is characterized by a wide array of persistent symptoms, including fatigue, cognitive dysfunction, respiratory complications, and cardiovascular issues, which can significantly impair quality of life and persist for months after the initial infection [4]. These prolonged cases not only represent a major clinical burden but may also serve as reservoirs for sustained viral replication and evolution, particularly in immunocompromised individuals. Such intra‐host viral persistence facilitates the accumulation of mutations that may increase viral fitness, immune escape, or transmissibility, potentially driving the emergence of new pathogenic strains [5, 6].

SARS‐CoV‐2 is a positive‐sense, single‐stranded RNA virus belonging to the Coronaviridae family, with one of the largest known RNA genomes (~ 30 kb) among RNA viruses [7]. The viral genome encodes 16 nonstructural proteins (Nsp1–Nsp16) essential for replication and transcription, along with four structural proteins: Spike (S), Envelope (E), Membrane/Matrix (M), and Nucleocapsid (N) [8]. These structural proteins are integral to viral assembly and infectivity, making them critical targets for antiviral drug development [9]. Among these, the N protein plays a pivotal role in assembling the viral RNA genome into a ribonucleoprotein (RNP) complex, and interacts with the M protein to facilitate the packaging of the RNP complex into nascent virions [10, 11]. Beyond its structural role, the N protein regulates viral transcription and replication and modulates host immune responses, including the suppression of interferon signaling [12, 13]. It interacts with multiple viral and host proteins, influencing key aspects of the viral life cycle, underscoring its potential as a therapeutic target [14]. Notably, the N protein is among the most abundant and highly conserved SARS‐CoV‐2 proteins, highlighting its functional significance.

The SARS‐CoV‐2 N protein consists of 419 amino acids and comprises two well‐characterized structural domains: an N‐terminal RNA‐binding domain (N‐NTD, residues 44–174) and a C‐terminal dimerization domain (N‐CTD, residues 247–365), while the rest of the protein is intrinsically disordered (Fig. 1) [15]. The N protein undergoes liquid–liquid phase separation (LLPS) in the presence of RNA, forming membraneless biomolecular condensates [16, 17]. While LLPS may facilitate virus assembly *in vivo*, heterologous expression systems often result in the formation of large, heterogeneous oligomeric assemblies due to interactions with cellular RNAs, complicating the purification of a homogeneous protein preparation for structural and functional studies. Several reports are available for purification from *E. coli* expression system with and without solubility tags [18, 19, 20, 21]. However, co‐purification of host cell RNA/DNA and formation of large oligomers have remained a major challenge. Several crystal structures of N‐NTD and N‐CTD are available in the Protein Data Bank [8, 15, 22]. However, a crystal structure or cryo‐EM structure of full‐length N protein is not yet available, likely due to challenges in obtaining high‐quality, stable protein preparations for structural studies. To address these purification challenges, here we report an optimized protocol to obtain soluble, RNA‐free SARS‐CoV‐2 N protein suitable for biophysical and structural studies, without using harsh chemical conditions or nucleases that may interfere with downstream applications. By incorporating a Thioredoxin (Trx) solubility tag and implementing a stringent chromatographic purification workflow, we have successfully isolated a highly pure, monodisperse N protein. A comprehensive suite of biophysical assays confirmed the protein's functional integrity, particularly its RNA‐binding activity. This study presents a refined purification strategy for the SARS‐CoV‐2 N protein, overcoming key challenges associated with its intrinsic disorder and biological condensate formation.

**Fig. 1:**
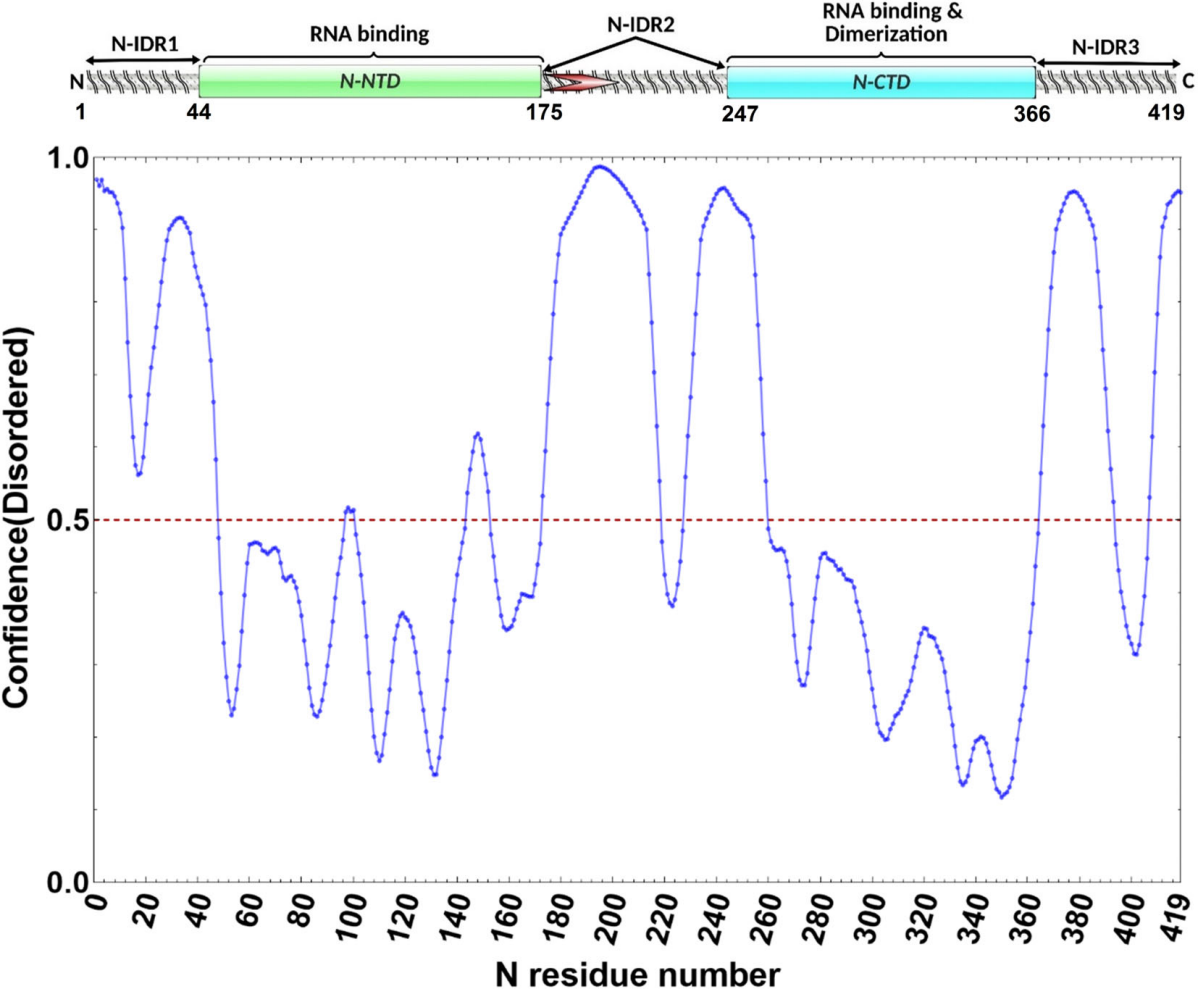
Domain architecture and intrinsic disorder prediction for the severe acute respiratory syndrome coronavirus 2 (SARS‐CoV‐2) Nucleocapsid (N) protein. The top schematic depicts the major functional regions of the N protein, including three intrinsically disordered regions (N‐IDR1, N‐IDR2, and N‐IDR3), the N‐terminal RNA‐binding domain (N‐NTD), and the C‐terminal dimerization domain (N‐CTD). A red arrow in N‐IDR2 highlights the SR‐rich region. The domain boundaries (residue numbers) have been marked. Below, the line graph shows the ‘Disorder profile plot’ for each residue (1–419). The values above threshold (0.5, the red dashed line) indicate a high probability of intrinsic disorder. This analysis highlights extensive disordered regions flanking the structured N‐NTD and N‐CTD, consistent with the flexible, multifunctional nature of the coronavirus N protein.

## Materials and methods

### Plasmid construction

The codon‐optimized gene encoding the full‐length SARS‐CoV‐2 N protein (UniProt ID P0DTC9) was chemically synthesized. The optimized gene sequence was PCR amplified using primer pair cat**ccatgg**agtctgataatggaccccaaaatc and cat**ggatcc**ttaggcctgagttgagtcag, and subcloned into the *NcoI* and *BamHI* sites of the *p*NH‐TrxT expression vector (Addgene plasmid #26106). This construct adds an N‐terminal 6xHis tag followed by a Thioredoxin (Trx) fusion partner and a TEV protease cleavage site upstream of the N protein. The plasmid insert was verified by Sanger DNA sequencing. The optimized gene and protein construct sequences are provided in Fig. S1. An additional glutamic acid (Glu) residue was introduced after the N‐terminal methionine as a result of cloning site artifacts.

### Expression and purification of Nucleocapsid protein

The N protein construct was expressed in *Escherichia coli* Rosetta™ (DE3) cells. Cultures were grown in LB medium with kanamycin (50 μg·mL^−1^) and chloramphenicol (25 μg·mL^−1^) at 37 °C until OD_600_ reached 0.8. Protein expression was induced with 0.25 mm IPTG, and cultures were grown overnight at 18 °C. Cells were harvested and re‐suspended in lysis buffer (25 mm Tris–HCl pH 8.0, 600 mm NaCl, 25 mm imidazole, 10% glycerol) with lysozyme (0.5 μg·mL^−1^) and PMSF (1 mm) as a protease inhibitor, followed by cell lysis using ultrasonication. The lysate was clarified by centrifugation (15 000 × ***g***, 45 min, 4 °C). The 6xHis‐Trx‐tagged N protein was purified via immobilized metal affinity chromatography (IMAC) using a Ni‐IDA sepharose column (GE Healthcare, Sweden) equilibrated in buffer H1 (25 mm Tris–HCl pH 8.0, 600 mm NaCl, 25 mm imidazole). Elution was performed in buffer H2 (25 mm Tris–HCl pH 8.0, 100 mm NaCl with a linear imidazole gradient (25 mm–1 m)). IMAC fractions were pooled and directly loaded on anion‐exchange chromatography column (Q Sepharose; GE Healthcare), pre‐equilibrated with buffer A1 (25 mm Tris–HCl pH 8.0, 100 mm NaCl). The N protein, free from nucleic acid contamination, was eluted with a linear gradient of NaCl (0.1–1 m). For tag removal, 6xHis‐TEV protease digestion was performed overnight at 4 °C. The cleavage mixture was passed through a Ni‐IDA column to remove His‐tagged components and the 6xHis‐TEV protease. The tag‐free N protein was collected in the flow‐through. To achieve final polishing and evaluate the oligomeric state, the N protein was subjected to size‐exclusion chromatography (SEC) on a Superdex 200 column (GE Healthcare) equilibrated with SEC buffer (25 mm sodium phosphate pH 7.0, 100 mm NaCl). The column was precalibrated with standard proteins (Alcohol dehydrogenase, 150 kDa; BSA, 66 kDa; Carbonic anhydrase, 29 kDa; Cytochrome C, 12.4 kDa). The N protein eluted as a single major peak; the corresponding fractions were pooled and used for subsequent analyses. The protein purity was analyzed after every step using SDS/PAGE. The protein was concentrated by ultrafiltration (30 kDa MWCO), and its concentration was determined by UV absorbance at 280 nm using the calculated extinction coefficient (ExPASy ProtParam). In an alternate purification approach, the N protein was purified without using anion‐exchange chromatography. Instead, protein purified using IMAC was treated with RNase A (10 μg·mL^−1^) to degrade the co‐purified RNA. The protein was incubated with RNase A and TEV protease overnight at 4 °C. The cleaved products were further purified by IMAC, followed by SEC, as mentioned above. The peak fraction corresponding to the dimer of N protein was used for assessing the RNA‐binding affinity of the protein.

### Dynamic light scattering

A Malvern ZetaSizer Nano‐ZS analyser (Malvern Instruments, UK) was used to conduct dynamic light scattering (DLS) measurements. The Nucleocapsid protein samples were first adjusted with SEC buffer to reach a concentration of 1 μg·mL^−1^. To ensure the removal of any particulate matter, the samples were centrifuged at 20,000 × ***g*** for 10 min prior to analysis. The measurements were performed at room temperature in a disposable polystyrene cuvette. For each sample, DLS spectra were obtained by performing three separate runs, with each run consisting of 12 individual 10‐s scans. These runs were then averaged to yield a final spectrum.

### Electrophoretic mobility shift assay

Electrophoretic mobility shift assay (EMSA) was performed to assess the RNA‐binding activity of the N protein using a 44 base long RNA (5′‐CUGUGUGGCUGUCACUCGGCUGCAUGCUUAGUGCACUCACGCAG‐3′) probe, corresponding to 5′ UTR of the virus genome, synthesized using an *in vitro* transcription kit. The RNA (~ 0.7 μm) was incubated with increasing concentrations of N protein in a 10 μL reaction containing SEC buffer. The mixtures were incubated at 4 °C for 30 min and resolved on a 1.5% agarose gel prestained with ethidium bromide. Gel electrophoresis was carried out in 1× TAE buffer, and RNA bands were visualized under UV light. A shift in the mobility of the RNA band upon addition of N protein indicated RNA‐protein complex formation.

### Fluorescence polarization binding assay

Fluorescence polarization assays were used to quantify the binding affinity of N protein for RNA. A fluorescein (FAM)‐labeled RNA probe (CGGUUUCGUCCGUGU, 20 nm final concentration) was incubated with varying concentrations of N protein (0 to 5 μm) in 100 μL SEC buffer in black 96‐well microplates. After incubation at room temperature for 30 min, fluorescence polarization (excitation 485 nm and emission 528 nm) was measured using a microplate reader (CLARIOstar; BMG Labtech, Germany). The polarization values (in millipolarization units, mP) were converted to fluorescence anisotropy values using system‐provided software. The fluorescence anisotropy values were plotted against protein concentration, and the data were fit to a one‐site binding model using nonlinear regression (GraphPad Prism) to determine the equilibrium dissociation constant (*K*_d_). All measurements were performed in triplicate, and results are reported as mean ± standard deviation.

## Results

### Purification of SARS‐CoV‐2 Nucleocapsid protein

**Fig. 2:**
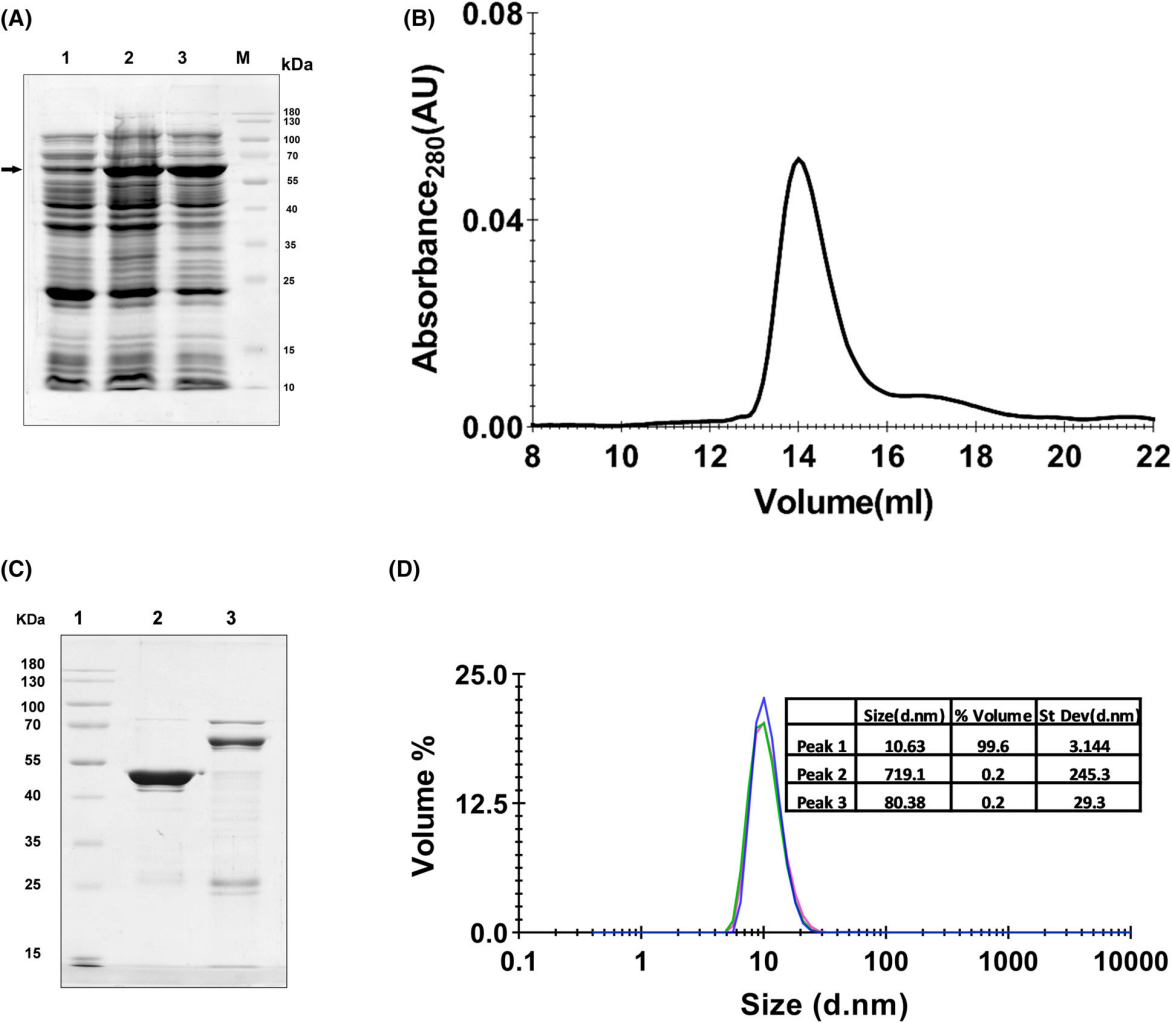
Purification of severe acute respiratory syndrome coronavirus 2 (SARS‐CoV‐2) Nucleocapsid (N) protein. (A) Coomassie‐stained SDS/PAGE analysis showing induction and soluble expression of Thioredoxin (Trx)‐tagged N protein. Lane 1, Uninduced cell lysate; Lane 2, Induced whole cell lysate; Lane 3, soluble fraction of cell lysate; Lane M, molecular weight marker. A prominent band corresponding to Trx‐tagged N protein (indicated by an arrow) is observed in both Lanes 2 and 3, indicating that the majority of the expressed N protein is present in the soluble fraction. (B) Analytical size‐exclusion chromatography (SEC) profile of the N protein, demonstrating its homogeneity in solution. The protein elutes as a single, symmetric peak at an elution volume of 14 mL, corresponding to an apparent molecular mass of ~ 90 kDa consistent with a dimeric N protein. (C) Coomassie‐stained SDS/PAGE analysis of the purified N protein. Lane 1: Molecular weight marker (kDa); Lane 2: Cleaved Nucleocapsid protein after four‐stage purification; Lane 3: Nucleocapsid protein with N‐terminal Trx tag, after IMAC and AEX purification, before TEV cleavage. The purity of the protein (Lane 2) was estimated to be > 96% based on the relative band intensities using imagej software. (D) Dynamic light scattering (DLS) analysis of purified N protein. More than 99% of the population is observed in the single major peak.

Nucleocapsid protein of SARS‐CoV‐2 contains three intrinsically disordered regions (IDRs): one at the N terminus, one at the C terminus, and a disordered linker region connecting N‐NTD and N‐CTD (Fig. 1). The disorder propensity of the protein was calculated using the *PrDOS* web server (https://prdos.hgc.jp/cgi‐bin/top.cgi) (Fig. 1). Initial expression trials revealed that the full‐length N protein tended to form inclusion bodies when overexpressed in *E. coli* (data not shown). To obtain soluble protein, we screened several fusion tags and found that an N‐terminal Thioredoxin tag significantly improved the expression of the soluble fraction of the N protein, as nearly all the expressed N protein was observed in soluble fractions of the cell lysate (Fig. 2A). The 6xHis‐Trx–N fusion was purified from the soluble cell lysate using IMAC (Fig. S2). However, after this first step, a substantial amount of nucleic acid was observed to co‐purify with the N protein, as evidenced by an elevated A_260/280_ ratio and an intense protein–RNA complex band on agarose gels (Fig. S2). This occurred despite using high‐salt lysis conditions, suggesting that the N protein binds nucleic acids very tightly. The bound nucleic acid was found to be RNA only (Fig. S2E). Treatment of the IMAC purified protein with RNase A did not successfully remove the contamination because a fraction of RNA appeared to remain tightly bound and protected from degradation, as evident from the higher A_260/280_ ratio (Table 1). We therefore employed an anion‐exchange chromatography (AEX) step to remove the bound nucleic acids. Notably, despite the high basic isoelectric point (pI ~ 10) of the N protein, the acidic Thioredoxin fusion tag facilitated the efficient binding of the N protein to the AEX column. The Trx–N fusion protein eluted at approximately 350 mm NaCl, whereas the RNA remained bound and eluted at a very high‐salt concentration, effectively separating the two (Fig. S2). After this step, the N protein was obtained free of any nucleic acid contamination (Table 1). Following purification, the N‐terminal tag was cleaved off, leaving only a single additional tag residue (Ser) at its N terminus (Fig. S1). The final purified N protein was highly homogeneous, eluting as a single sharp peak on SEC (Fig. 2B) consistent with a monodisperse and properly folded species. The peak corresponds to the dimer of N protein. The overall yield was nearly 25 mg of pure N protein from one liter of cell culture. In contrast, the protein purified without AEX exhibited a prominent peak at an earlier elution volume, indicative of a high molecular weight species consisting of an N–RNA complex (Fig. S3). A very small peak (not well resolved) was observed at the dimeric position; this fraction was used for EMSA and fluorescence polarization assays for comparison purposes. SDS/PAGE analysis confirmed the purity and the expected size of the tag‐cleaved N protein (Fig. 2C). Furthermore, DLS analysis (Fig. 2D) revealed that the purified N protein consists of one major population (> 99%), with a hydrodynamic radius of ~ 10.63 ± 3.1 nm and a polydispersity index (PDI) of 0.4, further attesting to the high purity and homogeneity of the sample. This higher PDI might be due to the inter‐domain movements, connected through an intrinsically disordered linker region.

**Table 1:** Comparison between absorbance ratios at 260 nm to 280 nm (A_260/280_).

Purification method (N protein)	Absorbance ratio (A_260/280_ nm)
Without anion‐exchange chromatography (RNase used to remove RNA)	0.861
With anion‐exchange chromatography	0.522

### RNA‐binding assessment using EMSA

**Fig. 3:**
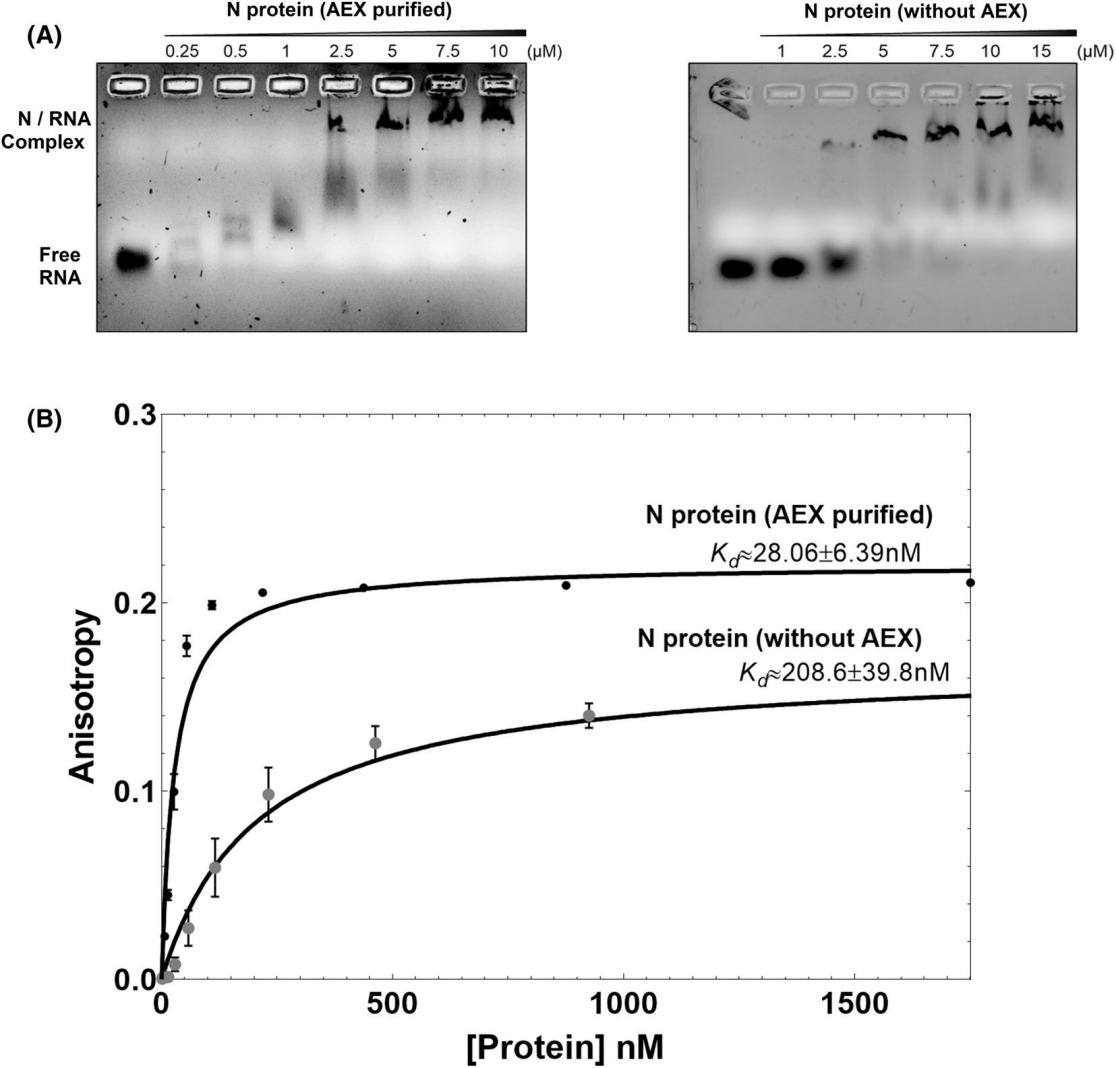
RNA‐binding studies of severe acute respiratory syndrome coronavirus 2 (SARS‐CoV‐2) Nucleocapsid (N) protein purified with or without anion‐exchange chromatography (AEX). (A) Electrophoretic mobility shift assay (EMSA) analysis, A 44‐nt viral RNA probe was incubated with increasing concentrations of N protein (0.25–10 μm, left panel; 1–15 μm, right panel). The protein purified with AEX exhibits strong RNA binding at submicromolar concentrations, as evidenced by the near complete shift of the unbound RNA band into a slower‐migrating N–RNA complex at 0.25 μm. In contrast, the preparation without AEX requires higher protein concentrations to achieve similar binding, reflecting its lower apparent affinity due to residual RNA contamination. (B) Fluorescence polarization assays. A FAM‐labeled 15‐mer SARS‐CoV‐2 UTR RNA (20 nm) was titrated with increasing concentrations of N protein. The protein sample free of contaminating RNA exhibited a markedly higher affinity (*K*_d_ = 28.0 ± 6.4 nm) compared with the sample retaining residual RNA (*K*_d_ = 208.6 ± 39.8 nm). Data points represent mean ± S.D. from at least three independent experiments, and solid curves depict best‐fit single‐site binding models. These findings underscore the critical role of eliminating bound RNA to accurately characterize the intrinsic RNA‐binding properties of the N protein.

The nucleic acid‐binding properties of the purified N protein were investigated using EMSA. The results revealed that the N protein exhibits strong RNA‐binding activity. Increasing concentrations of the N protein led to a progressive decrease in the mobility of the 44‐nucleotide RNA probe, indicative of complex formation. At 0.25 μm N protein, the free RNA band was nearly undetectable, suggesting that even submicromolar concentrations of the N protein are sufficient for RNA binding and sequestration (Fig. 3A). At 2.5 μm N protein, a distinct retarded band corresponding to the N–RNA complex became clearly visible, confirming stable complex formation. On the contrary, the protein purified using an alternative protocol without AEX, involving RNase A to remove the bound RNA, showed a comparatively weaker binding. A complete shift of the RNA band was observed at 5 μm protein concentration (Fig. 3A). Collectively, these EMSA results qualitatively demonstrate that the recombinant N protein retains strong affinity to RNA, and a complete RNA‐free preparation is required for true estimation of binding affinities.

### Affinity measurement of Nucleocapsid protein with RNA

Fluorescence polarization binding assays were conducted to determine the binding affinity of the N protein for RNA, comparing preparations purified with and without anion‐exchange chromatography. Notably, the N protein purified using anion‐exchange chromatography exhibited a significantly higher RNA‐binding affinity (Fig. 3B). The dissociation constant (*K*_d_) for its interaction with the fluorescent RNA probe was 28.1 ± 6.4 nm, whereas the N protein purified without anion‐exchange chromatography displayed a markedly weaker affinity (*K*_d_ = 208.6 ± 39.8 nm).

This more than sevenfold reduction in binding affinity is likely attributable to nonspecific RNA contaminants occupying RNA‐binding sites or promoting aggregation, thereby hindering probe interaction. Incorporating an anion‐exchange chromatography step effectively removed these contaminants, yielding an RNA‐free N protein preparation that accurately reflects its nanomolar binding affinity. These findings underscore the critical importance of stringent purification strategies, as only the RNA‐free N protein exhibited the expected high‐affinity interaction.

## Discussion

In this work, we have established a purification approach that yields full‐length SARS‐CoV‐2 N protein devoid of nucleic acid, enabling detailed biochemical characterization. The intrinsically disordered regions (nearly 40% of the protein is IDR) and propensity to form RNA‐induced LLPS and biological condensates hinder the recombinant expression and production of a homogenous protein preparation suitable for high‐resolution structure studies [23]. Various strategies have been explored to obtain RNA‐free N protein, which include maintaining high‐salt concentration during cell lysis, using ribonuclease during purification, or denaturation and renaturation of the protein [18, 19, 20]. Brudenell et al. have shown the isolation of RNA‐free protein using high‐salt washes (up to 2 m NaCl) during IMAC and using DNA/RNA precipitating agents [21]. In our study, we have employed a Trx fusion tag at the N terminus of the N protein, and the majority of the protein was observed in the soluble fraction of the cell lysate (Fig. S1). The Trx tag is widely used to improve the expression and solubility of recombinant proteins, especially those with large IDRs and those prone to forming inclusion bodies [24]. We found that conventional purification methods (even with high‐salt washes) were insufficient to remove all nucleic acid contaminants. Nuclease treatment alone was ineffective in our hands, as RNAs bound within N protein oligomers were protected from enzymatic digestion (Fig. S2E) [18]. A recent report has shown that treating N protein with RNase A altered the oligomeric state of the protein [25]. Additionally, any residual RNase could alter the outcome of downstream experiments involving RNA. We have included an anion‐exchange chromatography step to remove the bound RNA. The acidic Trx tag (pI ~ 5.5) facilitated the efficient binding of the highly basic N protein (pI ~ 10) to AEX column, which could efficiently separate the bound RNA. The Trx tag could easily be removed using TEV cleavage to get a nearly native N protein with three additional amino acids at N terminus. The result of this purification strategy is a highly pure, monodisperse N protein preparation. Size‐exclusion chromatography and DLS analysis confirmed that the RNA‐free N protein exists predominantly as a single species in solution, likely a dimer. In contrast, N protein purified with RNA attached forms large oligomeric aggregates (Fig. S3). Earlier studies showed that RNA‐bound N protein elutes in the void volume of SEC columns, while RNA‐free protein shifts to a lower oligomeric state, primarily dimers [23]. Our findings align with these observations, demonstrating that removing adventitious RNA profoundly impacts the protein's biochemical properties. Studies have also shown that RNA‐free SARS‐CoV‐2 N protein exhibits altered phase separation behavior, underscoring the importance of eliminating nucleic acid contaminants. Our data also show that RNA contamination influences the RNA binding on EMSA as well as in the fluorescence polarization assay (Fig. 3). Achieving an RNA‐free, homogeneous N protein preparation is crucial for structural studies. To date, high‐resolution structures of full‐length coronavirus N proteins are not available. Structural data for individual domains (NTD and CTD) exist, but understanding their arrangement in full‐length N protein requires a stable, monodisperse sample. Our purified N protein meets this criterion, being free of RNA and other heterogeneities that could cause aggregation or compositional variability. This makes it an excellent candidate for crystallization trials or cryo‐EM grid preparation. The availability of such a sample paves the way for determining the three‐dimensional architecture of the full N protein or its oligomeric complexes with Membrane/Matrix or other viral proteins, shedding light on genome packaging and interactions with other viral components. Beyond facilitating structural biology, our work has implications for antiviral discovery. The N protein has garnered attention as a potential antiviral target due to its essential role in the virus life cycle. Small‐molecule inhibitors that interfere with N protein function could prevent proper ribonucleoprotein (RNP) assembly or block interactions with viral RNA and the M protein.

In summary, we have developed a robust method to obtain SARS‐CoV‐2 N protein in a soluble, RNA‐free form, demonstrating that it retains high‐affinity RNA binding. This work overcomes a major challenge in biochemical and structural studies of the coronavirus N protein. With a purified and well‐behaved N protein, researchers can now pursue high‐resolution structural characterization and screen for small‐molecule inhibitors with greater confidence. Our findings highlight the importance of thorough purification in studying RNA‐binding proteins and provide a foundation for future investigations into the structure, dynamics, and inhibition of the SARS‐CoV‐2 N protein.

## Conflict of interest

The authors declare no conflict of interest.

## Peer review

The peer review history for this article is available at https://www.webofscience.com/api/gateway/wos/peer‐review/10.1002/2211‐5463.70064.

## Author contributions

SS contributed to the investigation, data analysis, and manuscript writing. GDG contributed to the conceptualization, data analysis, and manuscript writing.

## Supporting information


Data S1.

## Data Availability

The data that support the findings of this study are available in the methods and Supporting Informationl of this article. Additional information/data, if any, are available upon request from the corresponding author.

## References

[1] Wang W , Xu Y , Gao R , Lu R , Han K , Wu G and Tan W (2020) Detection of SARS‐CoV‐2 in different types of clinical specimens. JAMA 323, 1843–1844. 32159775 10.1001/jama.2020.3786 PMC7066521

[2] Chen X , Chen Z , Azman AS , Sun R , Lu W , Zheng N , Zhou J , Wu Q , Deng X , Zhao Z *et al*. (2022) Neutralizing antibodies against severe acute respiratory syndrome coronavirus 2 (SARS‐CoV‐2) variants induced by natural infection or vaccination: a systematic review and pooled analysis. Clin Infect Dis 74, 734–742. 34302458 10.1093/cid/ciab646 PMC9016754

[3] Davis HE , McCorkell L , Vogel JM and Topol EJ (2023) Long COVID: major findings, mechanisms and recommendations. Nat Rev Microbiol 21, 133–146. 36639608 10.1038/s41579-022-00846-2 PMC9839201

[4] Al‐Aly Z , Davis H , McCorkell L , Soares L , Wulf‐Hanson S , Iwasaki A and Topol EJ (2024) Long COVID science, research and policy. Nat Med 30, 2148–2164. 39122965 10.1038/s41591-024-03173-6

[5] Kemp SA , Collier DA , Datir RP , Ferreira IATM , Gayed S , Jahun A , Hosmillo M , Rees‐Spear C , Mlcochova P , Lumb IU *et al*. (2021) SARS‐CoV‐2 evolution during treatment of chronic infection. Nature 592, 277–282. 33545711 10.1038/s41586-021-03291-y PMC7610568

[6] Choi B , Choudhary MC , Regan J , Sparks JA , Padera RF , Qiu X , Solomon IH , Kuo HH , Boucau J , Bowman K *et al*. (2020) Persistence and evolution of SARS‐CoV‐2 in an immunocompromised host. N Engl J Med 383, 2291–2293. 33176080 10.1056/NEJMc2031364 PMC7673303

[7] von Delft A , Hall MD , Kwong AD , Purcell LA , Saikatendu KS , Schmitz U , Tallarico JA and Lee AA (2023) Accelerating antiviral drug discovery: lessons from COVID‐19. Nat Rev Drug Discov 22, 585–603. 37173515 10.1038/s41573-023-00692-8 PMC10176316

[8] Arya R , Kumari S , Pandey B , Mistry H , Bihani SC , Das A , Prashar V , Gupta GD , Panicker L and Kumar M (2021) Structural insights into SARS‐CoV‐2 proteins. J Mol Biol 433, 166725. 33245961 10.1016/j.jmb.2020.11.024 PMC7685130

[9] Kumari S , Mistry H , Bihani SC , Kumar M and Gupta GD (2020) Structural proteins of SARS‐CoV‐2 and assembly of new virions in the host cell. SMC Bulletin 11, 52–63. https://www.smcindia.org/pdf/SMC‐Bulletin‐August%202020.pdf

[10] Wu W , Cheng Y , Zhou H , Sun C and Zhang S (2023) The SARS‐CoV‐2 Nucleocapsid protein: its role in the viral life cycle, structure and functions, and use as a potential target in the development of vaccines and diagnostics. Virol J 20, 6. 36627683 10.1186/s12985-023-01968-6 PMC9831023

[11] Lu S , Ye Q , Singh D , Cao Y , Diedrich JK , Yates JR 3rd , Villa E , Cleveland DW and Corbett KD (2021) The SARS‐CoV‐2 Nucleocapsid phosphoprotein forms mutually exclusive condensates with RNA and the membrane‐associated M protein. Nat Commun 12, 502. 33479198 10.1038/s41467-020-20768-y PMC7820290

[12] Cong Y , Ulasli M , Schepers H , Mauthe M , V'kovski P , Kriegenburg F , Thiel V , de Haan CAM and Reggiori F (2020) Nucleocapsid protein recruitment to replication‐transcription complexes plays a crucial role in Coronaviral life cycle. J Virol 94, e01925. 31776274 10.1128/JVI.01925-19 PMC6997762

[13] Mu J , Fang Y , Yang Q , Shu T , Wang A , Huang M , Jin L , Deng F , Qiu Y and Zhou X (2020) SARS‐CoV‐2 N protein antagonizes type I interferon signaling by suppressing phosphorylation and nuclear translocation of STAT1 and STAT2. Cell Discov 6, 65. 32953130 10.1038/s41421-020-00208-3 PMC7490572

[14] Peng Y , Du N , Lei Y , Dorje S , Qi J , Luo T , Gao GF and Song H (2020) Structures of the SARS‐CoV‐2 Nucleocapsid and their perspectives for drug design. EMBO J 39, e105938. 32914439 10.15252/embj.2020105938 PMC7560215

[15] Kumari S , Mistry H , Bihani SC , Mukherjee SP and Gupta GD (2024) Unveiling potential inhibitors targeting the Nucleocapsid protein of SARS‐CoV‐2: structural insights into their binding sites. Int J Biol Macromol 273, 133167. 38885868 10.1016/j.ijbiomac.2024.133167

[16] Chau BA , Chen V , Cochrane AW , Parent LJ and Mouland AJ (2023) Liquid‐liquid phase separation of Nucleocapsid proteins during SARS‐CoV‐2 and HIV‐1 replication. Cell Rep 42, 111968. 36640305 10.1016/j.celrep.2022.111968 PMC9790868

[17] Jack A , Ferro LS , Trnka MJ , Wehri E , Nadgir A , Nguyenla X , Fox D , Costa K , Stanley S , Schaletzky J *et al*. (2021) SARS‐CoV‐2 Nucleocapsid protein forms condensates with viral genomic RNA. PLoS Biol 19, e3001425. 34634033 10.1371/journal.pbio.3001425 PMC8553124

[18] Di D , Dileepan M , Ahmed S , Liang Y and Ly H (2021) Recombinant SARS‐CoV‐2 Nucleocapsid protein: expression, purification, and its biochemical characterization and utility in serological assay development to assess immunological responses to SARS‐CoV‐2 infection. Pathogens 10, 1039. 34451501 10.3390/pathogens10081039 PMC8402198

[19] Li G , Li W , Fang X , Song X , Teng S , Ren Z , Hu D , Zhou S , Wu G and Li K (2021) Expression and purification of recombinant SARS‐CoV‐2 Nucleocapsid protein in inclusion bodies and its application in serological detection. Protein Expr Purif 186, 105908. 34048905 10.1016/j.pep.2021.105908 PMC8150265

[20] Wu C , Qavi AJ , Hachim A , Kavian N , Cole AR , Moyle AB , Wagner ND , Sweeney‐Gibbons J , Rohrs HW , Gross ML *et al*. (2021) Characterization of SARS‐CoV‐2 Nucleocapsid protein reveals multiple functional consequences of the C‐terminal domain. iScience 24, 102681. 34095780 10.1016/j.isci.2021.102681 PMC8168301

[21] Brudenell EL , Pohare MB , Zafred D , Phipps J , Hornsby HR , Darby JF , Dai J , Liggett E , Cain KM , Barran PE *et al*. (2024) Efficient overexpression and purification of severe acute respiratory syndrome coronavirus 2 Nucleocapsid proteins in *Escherichia coli* . Biochem J 481, 669–682. 38713013 10.1042/BCJ20240019 PMC11346444

[22] Dinesh DC , Chalupska D , Silhan J , Koutna E , Nencka R , Veverka V and Boura E (2020) Structural basis of RNA recognition by the SARS‐CoV‐2 Nucleocapsid phosphoprotein. PLoS Pathog 16, e1009100. 33264373 10.1371/journal.ppat.1009100 PMC7735635

[23] Tarczewska A , Kolonko‐Adamska M , Zarębski M , Dobrucki J , Ożyhar A and Greb‐Markiewicz B (2021) The method utilized to purify the SARS‐CoV‐2 N protein can affect its molecular properties. Int J Biol Macromol 188, 391–403. 34371045 10.1016/j.ijbiomac.2021.08.026 PMC8343380

[24] Mistry H and Gupta GD (2023) Transcription coupled DNA repair protein UVSSA binds to DNA and RNA: mapping of nucleic acid interaction sites on human UVSSA. Arch Biochem Biophys 735, 109515. 36623745 10.1016/j.abb.2023.109515

[25] Ribeiro‐Filho HV , Jara GE , Batista FAH , Schleder GR , Costa Tonoli CC , Soprano AS , Guimarães SL , Borges AC , Cassago A , Bajgelman MC *et al*. (2022) Structural dynamics of SARS‐CoV‐2 Nucleocapsid protein induced by RNA binding. PLoS Comput Biol 18, e1010121. 35551296 10.1371/journal.pcbi.1010121 PMC9129039

